# Treatment rationale and design of a phase II study of narrow-band ultraviolet B phototherapy for cutaneous steroid-refractory acute graft-vs-host disease following allogenic stem-cell transplantation

**DOI:** 10.1097/MD.0000000000016372

**Published:** 2019-07-12

**Authors:** Jun Asai, Junko Yamaguchi, Taku Tsukamoto, Yoshiaki Chinen, Yuji Shimura, Tsutomu Kobayashi, Norito Katoh, Junya Kuroda

**Affiliations:** a Department of Dermatology; b Division of Hematology and Oncology, Kyoto Prefectural University of Medicine Graduate School of Medical Science, Kyoto, Japan.

**Keywords:** allogenic hematopoietic stem-cell transplantation, biomarker, cutaneous acute graft-vs-host disease, efficacy, narrow-band ultraviolet B phototherapy

## Abstract

**Background:**

Acute graft-vs-host disease (aGVHD) is a common complication of allogenic hematopoietic stem-cell transplantation (allo-HSCT) and skin is the most common and often the 1st site at which aGVHD develops. Cutaneous aGVHD is usually treated with oral and/or topical corticosteroids as the 1st-line treatment; however, steroid-refractory aGVHD not only impairs patients’ quality of life but also causes significant morbidity and mortality after allo-HSCT. Narrow-band ultraviolet B (NB-UVB) phototherapy has been utilized for a wide range of immunologic inflammatory skin diseases, but there is limited information on the efficacy, safety, and biomarkers for response prediction of NB-UVB for cutaneous aGVHD.

**Aims:**

The purpose of this study is to investigate the efficacy and safety of NB-UVB phototherapy for steroid-refractory cutaneous aGVHD.

**Patients and methods:**

A total of 40 subjects aged from 16 to 70 years with steroid-refractory cutaneous aGVHD after allo-HSCT will be included in the trial. Patients with worse than stage 2 intestine/liver aGVHD will be excluded. Eligible patients will undergo NB-UVB phototherapy until resolution or further worsening of rash or occurrence of an unmanageable adverse event. The primary endpoint is the overall response rate. The secondary outcomes include rates for complete response, partial response, stable disease, progressive disease, duration of response, sparing effect on calcineurin inhibitors and/or corticosteroids, safety, and predictive biomarkers for treatment response.

**Ethics and dissemination:**

The protocol has been approved by the institutional Clinical Research Review Board of Kyoto Prefectural University of Medicine. Written informed consent will be obtained from all patients before registration, in accordance with the Declaration of Helsinki. Results of the study will be disseminated via publications in peer-reviewed journals.

**Trial registration:**

Trial registration numbers UMIN000032426 and jRCTs052180005.

## 1 Introduction

Allogenic hematopoietic stem-cell transplantation (allo-HSCT) is one of the best curative options for various malignant and nonmalignant hematologic disorders those are otherwise lethal. This is because this strategy includes not only the combined effects of intensive preparative cytotoxic treatment but also a potent graft-vs-tumor (GVT) effect through an allogeneic immune reaction with hematopoietic recovery by transfer of allogenic hematopoietic cells. However, transplanted immune cells also potentially cause immunologic attacks against normal tissues of recipients, and such so-called graft-vs-host disease (GVHD) may sometimes be severe.

Acute graft-vs-host disease (aGVHD), which normally affects the skin, liver, or gastrointestinal tract, accounts for 30% to 70% of major adverse events following allo-HSCT, despite prophylaxis with immunosuppressive agents.^[1]^ Corticosteroids are the frontline therapy for aGVHD, with response rates of approximately 40%.^[2]^ However, there is no promising salvage treatment for steroid-refractory (SR) aGVHD. Various systemic immunosuppressive interventions, such as use of antithymocyte globulin (ATG), immunosuppressive biologic agents, and mesenchymal stromal cells, have been used to treat SR-aGVHD, but their efficacies have not been satisfactory. Moreover, there is major concern regarding the excessive systemic immunosuppression induced by these therapies, which potentially increases the risk of infectious complications, disease recurrence, and emergence of secondary malignancy.^[1,3–7]^ In addition, ATG, biologic agents, and MSCs have high associated medical costs. Given this situation, SR-aGVHD is an important cause of morbidity and mortality as well as an economical issue after allo-HSCT.^[8,9]^ Thus, there is a high unmet medical need for establishment of effective and safe treatment for SR-aGVHD.

Skin is the most common and often the 1st site at which aGVHD develops, and the condition can be present as a single affected site or with multiple organ involvement. In general, cutaneous aGVHD initially manifests as a papular eruption that can be both focal and systemic, but when uncontrolled, it expands systemically and leads to desquamation resembling toxic epidermal necrosis. Thus, according to clinical stage and overall grading of aGVHD based on the affected skin area and the degree of other organ involvement, direct topical corticosteroids are commonly utilized for isolated grade I, stages 1 to 2 cutaneous aGVHD; while systemic corticosteroids are used as 1st-line salvage therapy for worse aGVHD of grades II to IV. However, even in a case with isolated cutaneous aGVHD without other organ involvement, more intensive systemic immunosuppressive therapy is still commonly used for SR-aGVHD. Recently, successful disease control of cutaneous aGVHD by ultraviolet-based phototherapy has been repeatedly reported in patients with skin involvement as the predominant feature of SR-aGVHD.^[10–13]^

Narrow-band ultraviolet B (NB-UVB) has a peak wavelength of 311 to 312 nm and excludes the shorter, photobiologically more erythrogenic wavelengths in UVB. Thus, it has been widely used as an effective and safe phototherapy for inflammatory skin diseases such as psoriasis, atopic dermatitis, and vitiligo vulgaris. With the assumption that the immunosuppressive effects of NB-UVB can be directed to skin, NB-UVB phototherapy is likely to impair systemic immune function.^[14]^ However, to date, NB-UVB phototherapy has not been approved for cutaneous aGVHD in Japan. Previous clinical studies of NB-UVB phototherapy for cutaneous aGVHD have included limited numbers of participants.^[10,11]^ Furthermore, although it is purported that NB-UVB therapy has fewer side effects and a lower long-term carcinogenic effect than those of psoralen plus ultraviolet,^[15]^ these properties have not been substantiated in patients treated by allo-HSCT. Therefore, we plan to investigate the efficacy and safety of NB-UVB phototherapy for cutaneous SR-aGVHD prospectively in the present study.

## 2 Methods

### 2.1 Study design

An open-label, single-center, nonrandomized, single-arm phase 2 intervention study will be conducted to examine the efficacy and safety of NB-UVB therapy for cutaneous SR-aGVHD. The protocol has been reviewed and approved by the institutional Clinical Research Review Board of Kyoto Prefectural University Hospital. Written informed consent will be obtained from patients before registration by research physicians in accordance with the Declaration of Helsinki. At least annual independent monitoring will be planned in accordance with Japanese clinical trial guidelines.

### 2.2 Endpoints

#### 2.2.1 Primary endpoint

The primary endpoint is the overall response rate (ORR), which is defined as the proportion of patients with a partial response (PR) or a complete response (CR).

#### 2.2.2 Secondary endpoints

The secondary endpoints are rates for CR, PR, stable disease (SD), and progressive disease (PD), duration of response (DoR), sparing effect on calcineurin inhibitors and/or corticosteroids, adverse events, profile of peripheral blood immune cells, including B cells, T cells, NK cells, CD4/CD8-positive T cells, and regulatory T cells, in association with the response to NB-UVB, and peripheral blood levels of inflammatory cytokines, such as interleukin (IL)-1, IL-8, and IL-17, in association with the response to NB-UVB.

### 2.3 Participants

The inclusion criteria are patients diagnosed with cutaneous SR-aGVHD, where “steroid-refractory” is defined as progression or no improvement after 5 consecutive days of topical or oral corticosteroids, or recurrence during tapering off of oral steroids; histologically confirmed cutaneous aGVHD (patients who do not undergo skin biopsy will only be included if the lesion is clearly diagnosed by the clinical course); and grades I to II aGVHD (stage 1, 2, or 3 cutaneous aGVHD and less than stage 1 intestinal/liver aGVHD); who are of age ≥16 to <70 years at the time of enrollment; have a performance status of 0 to 2 defined by Eastern Cooperative Oncology Group Criteria; provide written informed consent to participate in the study; are an outpatient or inpatient; and are of any gender.

The exclusion criteria are patients who are pregnant, nursing, or possibly pregnant; or have poorly controlled diabetes mellitus, uncontrolled infection, difficult-to-treat skin disease other than aGVHD, grade III or worse aGVHD, photosensitivity, or are judged to be inappropriate for the study by an attending physician.

### 2.4 NB-UVB phototherapy protocol

The NB-UVB phototherapy will be administered using a UV7001K-TL01 system (Waldmann-Lichttechnik, Villingen-Schwenningen, Germany) with peak emission at 311 to 313 nm (narrow band). Irradiation will be performed thrice a week in general; however, the frequency can be reduced to once or twice a week based on the severity of cutaneous aGVHD. Before irradiation, the minimal erythema dose (MED) will be determined by exposing different areas of each patient's upper back to a range of NB-UVB doses to identify the lowest dose that produces erythema within 24 hours. Initial NB-UVB will be started at 70% of MED and increased in steps of 20% up to 4-times MED or 120 times. In a case with development of slight erythema, treatment will be continued at the same dosage. In a case with erythema with pain or bulla formation, treatment will be withheld until erythema or bulla resolves and the dose will be reduced by 50% in resumed treatment. NB-UVB will be discontinued if the patient achieves CR, PD, NC, or continuous PR without further improvement. In a case with emergence of intestinal/liver aGVHD or worsening of coexisting intestinal/liver aGVHD during NB-UVB, the study treatment will also be discontinued.

### 2.5 Data collection

We will collect the following information in screening prior to the 1st NB-UVB irradiation: age, sex, diagnosis of hematologic disease and disease status, clinical aGVHD stage and grade, physical examination data, standard laboratory test results, and efficacy of concomitant aGVHD medication. Treatment visits, which will include a physical examination, clinical aGVHD grading, standard laboratory tests, evaluation of concomitant medication for aGVHD, and assessment of adverse events, will be performed at least weekly. aGVHD will be graded according to consensus criteria.^[16]^ CR is defined as the complete resolution of rash, PR as a decrease in cutaneous aGVHD stage of at least 1 level, and PD as worsening of the skin aGVHD stage. NC indicates nonresponders. Adverse events will be evaluated using National Cancer Institute Common Toxicity Criteria (CTCAE) ver. 4.0. In addition, protocol-specified immunophenotypic analysis will be performed to calculate the absolute numbers of peripheral blood immune cells (i.e., B cells, T cells, NK cells, CD4/CD8-positive T cells, and regulatory T cells) and serum cytokine analysis for IL-1, IL-8, and IL-17 at 3 points: within 7 days prior to the 1st NB-UVB irradiation, at 2 months from the start of NB-UVB, and at the end of the treatment.

### 2.6 Rationale for the setting of the number of enrolled participants

All participants enrolled in the study (full analysis set, FAS); that is, subjects excluding patients with serious violations (such as a serious protocol deviation and violation of inclusion/exclusion criteria) or revocation of consent from the FAS (per protocol set); and those among the FAS in which protocol treatment is provided at least once (safety analysis set) will be analyzed. By assuming that the threshold response rate is 50% and the expected response rate is 75% at a one-sided significance level α = 5% and a power of 1 − *β* = 80%, it is calculated that 25 patients are required for the 1st stage in Simon 2-stage theory.^[17]^ Assuming a dropout rate of 30% due to organ damage, complications after allo-HSCT, or relapse of underlying disease, and a 10% rate of inappropriate enrollments, we therefore set the target number of patients as 40.

### 2.7 Statistical analysis

1. ORR: a cumulative rate of CR rate and PR rate.
2. DoR: Time from documentation of response to NB-UVB to re-worsening of cutaneous aGVHD.
3. Sparing effect on calcineurin inhibitors and/or corticosteroids: Rate of patients in which the dose of calcineurin inhibitors and/or corticosteroids can be reduced after the initiation of NB-UVB.

### 2.8 Ethics

The trial received ethical approval from the Ethics Committee of Kyoto Prefectural University of Medicine, Kyoto, Japan (number: CRB5180001, the last edition ver. 2, August 10, 2018). The trial will be performed under the supervision and management of the Ethics Committee.

### 2.9 Trial status

Recruitment was opened in October 2018, and the planned last follow up will occur in March 2023. As of April 2019, 2 subjects have been enrolled.

## 3 Discussion and conclusion

With increases in the use of peripheral blood stem cells as a hematopoietic stem-cell source, HLA-mismatched and unrelated donors, and age of transplant recipients, GVHD will continue to be a serious challenge after allo-HSCT. Based on previous findings showing CR rates of 56% to 59% and ORR of 76% to 100%,^[10–12,15,18]^ NB-UVB may be a viable therapeutic option for cutaneous aGVHD that otherwise may need systemic intensive immunosuppressive treatment. Moreover, because NB-UVB targets the skin, it is likely to enable sparing of corticosteroids or other systemic immunosuppressants for aGVHD, and thereby reduce the risks for adverse events caused by systemic immunosuppression. Neither an effective therapeutic strategy for SR-aGVHD nor NB-UVB treatment for aGVHD has been approved in Japan. We expect this study to establish evidence for NB-UVB in management of cutaneous SR-aGVHD.

Adverse events will be cautiously monitored in the study, especially regarding relapse of underlying hematologic disease and emergence of secondary cutaneous malignancy. NB-UVB therapy suppresses the type 1 pathway (IL-12, interferon-γ, and IL-8), leads to apoptosis of skin-homing lymphocytes, increases the number of p53-positive epidermal cells, and reduces the number of Langerhans cells present in the epidermis and dermis.^[14,19]^ Iyama et al showed that NB-UVB treatment expanded CD4^+^CD25^+^Foxp3^+^ regulatory T cells in peripheral blood of patients with cutaneous aGVHD, and NB-UVB-induced regulatory T cells may be functional in the skin and intestine or liver, based on a mouse model of aGVHD.^[20]^ It is unknown if a systemic increase of regulatory T cells impairs the GVT effect and increases the risk for relapse,^[11]^ which emphasizes the need to be cautious about relapse of underlying hematologic malignancy. In addition, use of NB-UVB has occasionally been associated with emergence of cutaneous cancers, especially in immunocompromised patients, and GVHD itself may increase the risk of melanoma and nonmelanoma skin cancer.^[21,22]^ The cumulative dose of NB-UVB is generally lower for cutaneous aGVHD than for other dermatologic diseases, but the presence of skin cancer needs to be ruled out before starting treatment and emergence of skin cancer will be regularly monitored during and after NB-UVB with long-term follow-up.

In conclusion, this study is an open-label, single-center, nonrandomized, single-arm phase 2 study for patients with cutaneous SR-aGVHD. To the best of our knowledge, this is the largest clinical trial of NB-UVB therapy for cutaneous aGVHD. This study may provide new evidence for SR-aGVHD treatment after allo-HSCT.

## Acknowledgments

The authors thank the patients, their families, and all investigators involved in the study.

## Author contributions

**Conceptualization:** Jun Asai, Junko Yamaguchi, Taku Tsukamoto, Norito Katoh, Junya Kuroda.

**Data curation:** Jun Asai, Junko Yamaguchi, Taku Tsukamoto, Yoshiaki Chinen, Yuji Shimura, Tsutomu Kobayashi, Junya Kuroda.

**Formal analysis:** Jun Asai, Junko Yamaguchi, Taku Tsukamoto, Yoshiaki Chinen, Yuji Shimura, Tsutomu Kobayashi, Junya Kuroda.

**Funding acquisition:** Norito Katoh, Junya Kuroda.

**Investigation:** Jun Asai, Junko Yamaguchi, Taku Tsukamoto, Yoshiaki Chinen, Yuji Shimura, Tsutomu Kobayashi, Norito Katoh, Junya Kuroda.

**Methodology:** Jun Asai, Junko Yamaguchi, Norito Katoh, Junya Kuroda.

**Project administration:** Jun Asai, Norito Katoh, Junya Kuroda.

**Resources:** Norito Katoh, Junya Kuroda.

**Software:** Norito Katoh, Junya Kuroda.

**Supervision:** Norito Katoh, Junya Kuroda.

**Validation:** Norito Katoh, Junya Kuroda.

**Visualization:** Norito Katoh, Junya Kuroda.

**Writing – original draft:** Jun Asai, Junko Yamaguchi.

**Writing – review & editing:** Norito Katoh, Junya Kuroda.

Junya Kuroda orcid: 0000-0001-6130-1550.
